# Safety and feasibility of exercise interventions in patients with hematological cancer undergoing chemotherapy: a systematic review

**DOI:** 10.1007/s00520-023-07773-9

**Published:** 2023-05-15

**Authors:** Anja Großek, Karla Großek, Wilhelm Bloch

**Affiliations:** 1 Department of Molecular and Cellular Sports Medicine, Institute of Cardiovascular Research and Sports Medicine, German Sport University Cologne, Am Sportpark Müngersdorf 6, 50933 Cologne, Germany; 2 Department of Physiotherapy, Hogeschool Zuyd, Heerlen, the Netherlands

**Keywords:** Leukemia, Lymphoma, Physical activity, Feasibility, Safety

## Abstract

**Objective:**

Exercise during and after cancer treatment has established quality of life and health benefits. However, particularly for patients with hematological cancer clear recommendations regarding the safety and feasibility of exercise are under-investigated. The aim of our systematic review was to summarize the literature regarding the feasibility and safety of exercise interventions in patients diagnosed with hematological cancer undergoing chemotherapy.

**Method:**

A systematic literature review was conducted using PubMed, SPORTDiscus, MEDLINE, Science Direct, and Web of Science electronic databases. Eligible studies were scientific publications reporting the feasibility and/or safety of an exercise intervention program carried out in inpatient patients diagnosed with hematological cancer undergoing chemotherapy.

**Result:**

Out of 12 studies (six RCTs) included in this review, six investigations reported results with regard to safety and 10 with regard to feasibility. While all studies claim that their exercise interventions were safe and/or feasible, it is noteworthy that this claim often remains unsupported as detailed information on how the feasibility of the intervention was asserted is missing.

**Conclusion:**

Exercise appears to be safe and feasible in hematological cancer patients. However, due to a striking lack of information on how the feasibility of the intervention was asserted, contextualizing the results and deducing recommendations for further studies remains challenging. Further research should therefore incorporate information on the execution of the exercise intervention in more detail.

**Supplementary Information:**

The online version contains supplementary material available at 10.1007/s00520-023-07773-9.

## Introduction

Hematological malignancies, such as acute leukemia and aggressive lymphoma, are life-threatening and fast-progressing diseases which cause symptoms like anemia, vulnerability to infections, fever, bleeding, nausea, weight loss, and fatigue [1]. A curative treatment requires a rapid hospitalization and initial high-dose chemo- or induction therapies and comes along with multiple toxicities and extended bedrest [2, 3]. The symptoms caused by the disease, side effects of the treatment (e.g., cytopenia, depression, fatigue), and inactivity can lead to physical and mental deconditioning, which causes a decline in quality of life [4, 5].

A variety of exercise intervention studies have demonstrated a beneficial effect on both physical and psychological outcomes, even stating the need for the implementation of different exercise regimes into usual care in cancer patients [6–8]. This suggests that exercise might be as promising in patients diagnosed with acute leukemia or aggressive lymphoma as it is for patients diagnosed with breast, colorectum, or prostate cancer. Nevertheless, findings for solid tumors might not be applicable to systemic tumors. Furthermore, variations in systemic tumors need to be considered. Conclusions from solid tumors might apply differently to patients diagnosed with acute leukemia compared to those diagnosed with chronic leukemia since these patients underlie unequal prerequisites due to differences in the aggressiveness of their specific treatment (e.g., higher treatment-related mortality often caused by infections requiring high hygiene standards during training, extended bed-rest, lower blood counts, and multiple toxicities leading to physical deconditioning for patients with acute leukemia).

Proving the beneficial effect of exercise interventions, requires studies, which are specifically designed for hospitalized hematological patients under treatment.

The gold standard to test the effectiveness of an exercise intervention is a randomized controlled trial (RCT), which is often not only expensive but also organizationally demanding. To minimize the cost and maximize the success, it is essential to rule out any unexpected complications as best as possible prior to the start of the RCT. Feasibility studies are a useful method to test the RCT’s processes, as they question “whether a future trial can be done, should be done and if so, how” [9] before embarking on the actual RCT. While testing the RCT’s processes, a feasibility study can focus on a wide array of different areas such as acceptability, demand, implementation, practicality, adaptation, integration, expansion, and limited-efficacy testing [10]. Hence, feasibility studies can become complex studies themselves and therefore rarely cover all the potential disturbing factors simultaneously. Feasibility studies must not be confused with pilot studies, which are considered a subset of feasibility studies, but follow the study protocol of the future RCT on a smaller scale [9, 11] instead of focusing on testing the RCT’s processes.

To the knowledge of the authors, a variety of researchers already investigated the potential of exercise interventions in hematological patients. Nevertheless, participants were not necessarily diagnosed with such severe types as investigated in this study. Additionally, participants were in different stages of their therapy and the focus of these trials was mostly on the effectivity on the training program, not giving clear recommendations or suggestions with regard to the feasibility and challenges of the chosen exercise program [12–14].

To contribute to improving the scientific standard of future intervention studies, this paper aims to systematically review the current literature on feasibility studies of exercise and physical activity interventions in patients diagnosed with hematological malignancies. Additionally, it further investigates the study designs and aims of the included investigations and evaluates which influence they might have on the feasibility and safety assessment of the target group.

## Method

This systematic literature review was conducted in accordance with the PRISMA (Preferred Reporting Items for Systematic Reviews and Meta-Analyses) guidelines [15].

### Eligibility criteria

Only articles published in peer-reviewed scientific journals with full-text access in the English language were included. Further in- and exclusion criteria were defined using the PICOS (Population, Intervention, Comparison, Outcome, Study Design) scheme [16]. Population: The participants of the included studies needed to be older than 18 years and be currently diagnosed with acute leukemia or an aggressive form of lymphoma. Regardless of entity, the participants needed to be hospitalized to undergo induction or high-dose chemotherapy. Intervention: Studies were eligible for this review if they included any kind of exercise or physical activity intervention, which was scheduled next to the induction/high-dose chemotherapy. Studies were excluded if they primarily investigated behavioral interventions, or the exercise intervention was not supervised and only advised. Comparison: As this investigation focused primarily on the feasibility and safety of exercise interventions, a control group was less relevant. Outcome: Any reporting of feasibility was evaluated. Mentioning of the term ‘feasibility’ or ‘feasible’ sufficied to include the study in this review. Additionally, adherence, retention, and recruitment rates which are often related to feasibility were analyzed. Study design: To get the broadest view of the existing literature, randomized controlled trials were included alongside non-randomized controlled trials and uncontrolled trials.

### Search strategy

This systematic literature review was conducted by searching the following electronic databases for relevant literature: PubMed, SPORTDiscus, MEDLINE via EBSCOhost, Science Direct, and Web of Science. The search was completed by AG on April 10, 2022, and was not limited to the earliest publishing year. The detailed search strings are provided in Table 1.

**Table 1:** Search string for each database

Databases	Search strategy
PubMed	(((leukem*[Title/Abstract] OR leukaem*[Title/Abstract] OR lymph*[Title/Abstract] OR hematological[Title/Abstract]) AND (chemotherapy* OR induction therap*)) AND (exercise[Title/Abstract] OR training[Title/Abstract] OR sport*[Title/Abstract] OR resistance[Title/Abstract] OR aerobic[Title/Abstract] OR strength[Title/Abstract] OR walking[Title/Abstract] OR ergometer[Title/Abstract] OR physiotherap*[Title/Abstract] OR physical therap*[Title/Abstract] OR endurance[Title/Abstract] OR yoga[Title/Abstract] OR tai chi[Title/Abstract])) AND (feasib* OR attendance OR adherence OR retention OR recruitment OR pilot OR safety OR adverse events OR adverse effects)
SPORTDiscus	((leukem* OR leukaem* OR lymph* OR hematological) AB abstract) AND ((chemotherapy* OR induction therap*) TX All Text) AND ((exercise OR training OR sport* OR resistance OR aerobic OR strength OR walking OR ergometer OR physiotherapy* OR physical therap* OR endurance OR physical activity) AB abstract) AND feasib* OR attendance OR adherence OR retention OR recruitment OR pilot OR safety OR adverse events OR adverse effects) TX All Text)
MEDLINE	((leukem* OR leukaem* OR lymph* OR hematological) AB abstract) AND ((chemotherapy* OR induction therap*) TX All Text) AND ((exercise OR training OR sport* OR resistance OR aerobic OR strength OR walking OR ergometer OR physiotherapy* OR physical therap* OR endurance OR physical activity) AB abstract) AND feasib* OR attendance OR adherence OR retention OR recruitment OR pilot OR safety OR adverse events OR adverse effects) TX All Text)
Science direct	(Article (feasibility OR adherence OR pilot OR feasible OR safety) AND (chemotherapy OR induction therapy)) AND (Title, abstract or author-specified keywords (leukemia OR leukaemia OR lymphoma OR hematological) AND (exercise OR training OR sport OR physical activity OR resistance))
Web of science	(leukem* OR leukaem* OR lymph* or hemtological (Abstract)) AND (chemotherap* OR induction therap* (All Fields)) AND exercise OR training OR sport* OR resistance OR aerobic OR strength OR walking OR ergometer OR physiotherap* OR physical therap* OR endurance OR physical activity (Abstract)) AND (feasib* OR attendance OR adherence OR retention OR recruitment OR pilot (All fields))

The title and abstracts of all articles were screened according to the eligibility criteria by AG. The remaining full-text articles were subsequently reviewed for eligibility by AG and KG. Relevant information from the final publications was extracted independently by AG and KG and disagreements were solved through discourse.

### Data extraction

Interestingly, definitions of “feasibility” and “pilot study” are not as distinct as expected. Consequently, there is a large variety of possible study designs for investigations testing feasibility. To structure the search results, studies are divided into randomized pilot studies, non-randomized pilot studies, and other feasibility studies as suggested by Eldridge et al. [17]. To give a detailed and structured view of the different exercise interventions, they are arranged according to the FITT (frequency, intensity, time, and type of exercise) principles as this is a common procedure to describe exercise interventions in depth [18–22]. Although safety is also considered under feasibility aspects occasionally, it is described separately in this review to emphasize the importance of safe exercise interventions for an already vulnerable population. Both feasibility and safety are reported as in the investigated studies. For safety, the mere absence of adverse events suffices to consider a study safe. For feasibility additionally, recruitment rate, recruitment period, recruitment time rate, retention rate, and training participation were investigated. The recruitment rate was defined by the number of participants who signed the informed consent divided by the number of eligible patients who were screened [23, 24]. The recruitment period represents the time (in months) between the first and last recruitment. As described by Walters et al. [25], the first and last months were counted as a whole if not described differently. The recruitment time rate was calculated by the number of recruited participants per month [25]. Finally, the retention rate was determined by the number of participants attending the final assessment divided by participants randomized [26]. Finally, training participation was presented as adherence (attended exercise sessions divided by planned exercise sessions) or as training sessions per week.

## Results

**Fig. 1:**
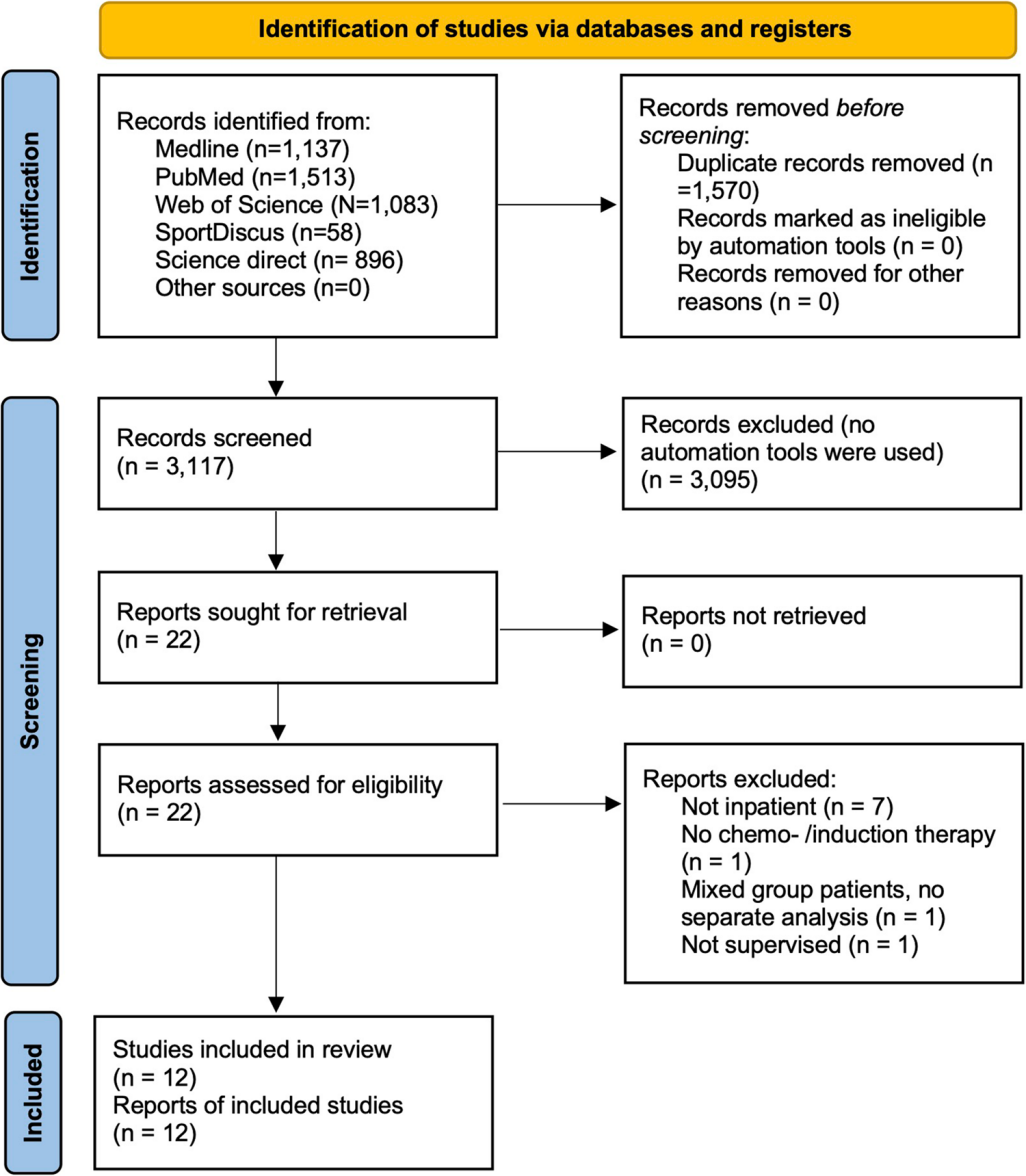
Flow diagram for literature search of the systematic review

The literature search executed on electronic databases yielded 4687 results, of which 3117 remained after the removal of duplicates. Of these, 12 met the inclusion criteria and were included in this systematic literature review. The full identification, screening, and inclusion process is presented in Fig. 1. All included studies consider themselves “pilot studies” or claim any kind of researched feasibility of their chosen activity intervention [8, 23, 24, 27–35], even if feasibility is neither a declared primary nor secondary outcome in some studies. The group of patients varies between patients diagnosed with acute myeloid leukemia (AML) only, acute leukemia, lymphoma only, and a mixed group of leukemia and lymphoma patients, which were investigated in five [23, 24, 27, 30, 33], two [29, 35], one [34], and four studies [8, 28, 31, 32], respectively. A more detailed overview of the study characteristics and interventions is presented in Table 2.

**Table 2:** Overview of the study characteristics

Author	Study type	Diagnosis and treatment	Age (years)	Intervention	Control	Adverse events	Recruitment rate, recruitment period, recruitment time rate	Retention rate	Training participation (adherence % or training per week)	Comments
Alibhai 2012 [24]	Non-randomized pilot study	Newly diagnosed AML or relapsed AML	56.4 (±12.9)	Individualized, supervised, mixed-modality exercise program (*n*=35)	No control group per design	One possible grade II musculoskeletal event occurred	67%; 9 months; 3.9 participants/month	97%	45.8%	
Alibhai 2015 [23]	Feasibility study	Newly diagnosed AML or relapsed AML	IG: 58 (±13.9) CG: 52 (15.8)	Individualized, supervised, mixed-modality exercise program, 4–6 weeks (*n*=57)	Usual care (*n*=24)	4 grade II musculoskeletal events (3 possible and 1 probably related to the intervention)	56%; 21 months; 3.9 participants/month	96%	54%	
Battaglini 2009 [27]	Feasibility study	Newly diagnosed AML or relapsed AML	35.7 (± 8.9)	Individualized prescriptive aerobic exercise program, 3–5 weeks (*n*=10) Mixed modality	No control group per design	Not reported	Not reported; not reported	80%	Not reported	Participants were excluded if they were older than 55 years
Baumann 2012 [28]	Non-randomized pilot study	Both groups: AML: 7 ALL: 2 HL: 3 NHL: 6	IG: 46.11 (±16.22) CG: 45.22 (±15.21)	Three weeks ergometer training (*n*=18)	Usual care (*n*=18)	Not reported	Missing information; 17 months; 2.1 participant/month	Not reported	Training per week: 2.4 (± 0.66) times, 25 (±7.9) minutes	
Bryant 2018 [29]	Randomized pilot study	IG: ALL: 1 AML: 7 CG: ALL: 1 AML: 8	IG: 52 (± 13) CG: 49 ( [15]	Individualized, supervised, mixed-modality exercise program, (*n*=9)	Usual care (*n*=9)	No adverse events occurred	48.6%; 14 months; 1.3 participants/month	94.4%	80% adherence of visits completed, with a mean of 6 sessions attended per week	Initial recruitment plan was for 30 patients, close early due to recruitment issues – that is, identifying patients without comorbidities
Chang 2008 [30]	Feasibility study	AML	IG: 49.4 (±15.3) CG: 53.3 (±13.6)	Supervised walking exercise program, 3 weeks (*n*=11)	5x/week non-invasive routine care by a research assistant, (*n*=11)	Not reported	85.7%; not reported	91.7%	Not reported	
Duregon 2019 [31]	Feasibility study	IG: AML:15 ALL:0 NHL: 5 HL: 7 MM: 3 CG: AML: 5 ALL:1 NHL:3 HL:2 MM:1	IG: 48.7 (±14.4) CG: 50.8 (±12.7)	Individualized, supervised mixed-modality exercise program (*n*=30)	Usual care (*n*=12)	Not reported	Not reported; 9 months; 4.7 participants/month	59.5%	Not reported	
Elter 2009 [32]	Non-randomized pilot study	AML: 7 ALL: 1 NHL: 4	44.16 (±13.85)	Ergometer training (*n*=12)	No control group per design/n.a.	Not reported	Not reported	66,67%	Not reported	
Klepin 2011 [33]	Non-randomized pilot study	AML	65.1 (±7.8)	Mixed-modality exercise program (*n*= 21)	No control group per design/n.a.	No adverse events occurred	43.6%; 19 months; 1.3 participants/month	52.4%	2.7 per week (range 0-8, ± 2.4),	Inclusion criteria: Age ≥ 50 years
Pahl 2018 [8]	Randomized pilot study	IG: AML: 1 ALL: 1 APL:1 NHL: 2 t-cell L: 1 CG: AML:4 MM:1	IG: 47 (19–62) CG: 56 (32–63)	Supervised whole body vibration (*n*=10)	Supervised aerobic exercise (*n*=10)	No adverse events occurred	28.6%; 7 months; 2.9 participants/month	55%	IG 62%; CG 67%	
Streckmann 2014 [34]	Feasibility study	Lymphoma patients	IG: 44 (20–67) CG: 48 (19–73)	36 weeks, supervised mixed-modality exercise program (*n*=30)	Usual care (*n*=31)	No adverse events are statistically and clinically meaningful	32.8%; 39 months; 1.6 participants/month	83.6%	65% (Highest for SMT, lowest for strength)	low recruitment-rate lead to end of study after three years not reaching the goal of 92 participants per group
Wehrle 2019 [35]	Randomized pilot study	AL	IGa: 47.7 (21.9–63.4) IGb: 47.4 (41.2–62.2) CG: 50.6 (35.0–58.1)	Endurance training (IGa, *n*=9) Resistance training (IGb: *n*=10)	3×/week low-intensity mobilization and stretching program (*n*=10)	No adverse events occurred	74.4% [1]; 33 months; 0.9 participants/month	75.9%	IGa: 68.9% IGb: 76.0% CG: 60.0%	Low recruitment, recruitment process was stopped after 2,5 years

*IG* intervention group, *CG* control group, *AML* acute myeloid leukemia, *ALL* acute lymphoblastic leukemia, *HL* Hodgkin lymphoma, *NHL* non-Hodgkin lymphoma, *MM* multiple myeloma, *t cell L* t cell lymphoma, [1] *based on calculation*; deviates from the publicized number by approx. 10%

### Study designs and aims

Six of the included studies followed a randomized controlled study design [8, 23, 29, 30, 34, 35], four had no control group [24, 27, 32, 33], one was a matched pair control [28], and one had a convenience sample [31] (patients, who chose to not participate in accordance with the exercise protocol, were asked to be part of the control group). It was further possible to classify all included studies into one of the following groups: randomized pilot studies [8, 29, 35], non-randomized pilot studies [24, 28, 32, 33], or feasibility studies [23, 27, 30, 31, 34]. This classification was done in accordance with the conceptual framework to define the feasibility and pilot studies of Eldridge et al. [17]. In a first step, the following sections will elaborate in more detail on the study design and aims of each of the studies per each classified group to contextualize one study’s results with the results from studies with a similar study design.

### Randomized pilot studies

Bryant et al. [29] performed a two-armed investigation with an intervention (mixed-modality; *n*=9) and a control (usual care, *n*=9) group. The study aims were to (i & ii) examine the effects of the intervention on a variety of physical and psychological health outcomes and (iii) evaluate the effect of the intervention on adherence to exercise. Pahl et al. [8] conducted a two-armed investigation with an intervention (whole body vibration (WBV), *n*=6) and an active control (cycling, *n*=5) group, to evaluate the following study aims: (i) feasibility of WBV by investigating training compliance, exercise-related adverse events, and a self-designed questionnaire; and (ii) effects on functional performance and mobility. Wehrle et al. [35] performed a three-armed trial with an endurance (*n*=9), a resistance (*n*=10), and a control (*n*=10) group. The study aim of this investigation was to investigate the independent effects of the two interventions on physical capacity and QOL.

### Non-randomized pilot studies

Out of the four non-randomized pilot studies, three [24, 32, 33] were designed without a control group. Alibahai et al. [24] (*n*= 35) aimed to (i) determine recruitment, retention, and ability to participate in the intervention; (ii) provide efficacy estimates on physical fitness outcome measures; (iii) examine the safety of the program; (iv) provide estimates on the effects of the intervention on QOL and fatigue; and (v) understand the impact of exercise on AML treatment tolerability. Elter et al. [32] (*n*=12) addressed the feasibility of the intervention as well as investigating any effect. Klepin et al. [33] (*n*=24) assessed (i) the feasibility of the intervention by investigating the recruitment rate, number of exercise sessions, percentage of participants completing assessments, and barriers to recruitment, as well as (ii) preliminary data on the efficacy of the training on different health outcomes. The fourth non-randomized pilot study conducted by Bauman et al. [28], following a matched-pair design, aimed to investigate (i) the effect of training interventions on the incidence of fever and pneumonias, (ii) changes in neutrophile- or leukocyte counts, and (iii) time spent in the hospital.

### Feasibility studies

Three studies [23, 30, 34] were identified as feasibility studies following a randomized controlled trial design. The phase II randomized controlled feasibility trial by Alibhai et al. [23] aimed to determine (i) the feasibility (via rates of recruitment, retention, and adherence), safety (by reporting adverse events), and preliminary evidence of the efficacy of the training program on health-related outcomes; and (ii) the impact of the intervention on treatment tolerability by investigating the length of stay, development of sepsis, intensive care unit admissions, and delays in further treatment. In their randomized controlled trial Chang et al. [30] aimed to develop and preliminarily examine the effects of a clinical feasible exercise intervention. Streckmann et al. [34] evaluated the effect of the training intervention on (i) QOL, (ii) peripheral neuropathy, (iii) activity levels, (iv) balance control on static surfaces, and (v) balance control on dynamic surfaces.

A two-armed trial with a convenience sample was executed by Duregon et al. [31]. They investigated (i) the feasibility of the exercise program, (ii) the implementation of the support of tablets in the exercise routine, and (iii) the effects of the training program on physical function. Battaglini et al. [27] examined in their one-armed study (i) the feasibility of the intervention and (ii) the effect of the intervention on health-related outcomes.

### Study Interventions by FITT-Criteria

This section summarizes the studies with regard to the FITT criteria to give an overview of the conducted training interventions. A mixed modality exercise program was investigated by seven studies [23, 24, 27, 29, 31, 33, 34], three investigated an endurance exercise program [28, 30, 32], one study compared whole body vibration against endurance training [8], and one study investigated endurance as well as resistance exercises in a three-armed trial [35]. The duration of the interventions varied between two and four weeks [8] and ranged up to 36 weeks [34]. Training frequencies ranged between two [34] and five [30, 31] times per week with a duration between 12 min [30] and 60 min [23, 34]. A more detailed overview of the frequencies, intensity, time, and type of exercise is presented in Table 3.

**Table 3:** FITT criteria of study interventions

Author	Frequency	Intensity	Time	Type of exercise	Comments
Alibhai 2012 [24]	4–5 times per week	Light to moderate intensity Aerobic exercise: RPE 3–6 (out of 10) or 50–75% of their heart rate reserve (HRR)	30–45 min Aerobic: 10–40 min Resistance 10–25 min Flexible training: 5–10 min	Mixed modality Aerobic exercise: walking or stationary cycling Resistance training: resistance bands and/or free weights, major muscle groups Flexible training: static stretching	HRR was found to be obstructive for patients due to intravenous lines ➔ was dropped Progression and adaptation: Aerobic: first increase in length and then in intensity Resistance: increasing the number of sets, repetitions, or resistance
Alibhai 2015 [23]	4–5 times per week	Light to moderate intensity Aerobic exercise: RPE 3-6 (out of 10) AND 50-75% of their heart rate reserve	30–60 min	Mixed modality Aerobic exercise: treadmill, hall walking, or stationary cycling Resistance training: body weight, resistance bands and/or free weights, major muscle groups Flexible training: static stretching	Time: depending on the participant’s ability and clinical symptoms Progression and adaptation: Aerobic: first increase in length and then in intensity Resistance: increasing the number of sets, repetitions, or resistance
Battaglini 2009 [27]	3–4 times per week (min. 36h rest between sessions)	Submaximal intensity Endurance: 40–50% HRR and RPE < 5 (out of 10) Resistance exercise: < 5 (out of 10)	30 min. each bout Light stretching: 3–5 min Endurance training: 5–10 min Resistance training: 5–15 min Core exercise: 5–10 min	Mixed modality Light stretching Endurance training: treadmill or stationary cycling Resistance training: dumbbells, resistance bands, and exercise ball Core exercise	Time: depended on the patient’s physical state on the day Each session was divided into two bouts, one in the morning and one in the afternoon
Baumann 2012 [28]	3 times per week	Moderate intensity RPE 13 (out of 20); max. HR 180 age; 80% max. HR	20–30 min	Endurance exercise: stationary cycling	
Bryant 2018 [29]	4 times a week,	Light to moderate intensity: Aerobic 50–70% HRR Resistance intensity increased using a 10 Rep. Max raining protocol	20–40 min Aerobic training: 5–15 min Resistance training: 10–20 min Stretching 5 min	Mixed modality Aerobic: walking or stationary cycling Resistance: resistance bands Stretching	Was adapted based on the patient’s physical limitations Twice a day
Chang 2008 [30]	5 times per week	Light intensity Target heart rate: resting heart rate plus 30	12 min	Hallway walk	
Duregon 2019 [31]	5 times per week	Light to moderate intensity RPE: 11–13 (out of 20)	15–30 min	Mixed modality Warm-up: breathing exercise and joint mobility Central part: proprioception, resistance (1–2 sets, with 8–101 repetitions and 12 min rest), and flexible training Cool down: relaxing and breathing	Additionally, workout autonomously, exercise program on a tablet) Performance and exercise volume could vary every day, based on the health conditions of patients During aplasia no strength training
Elter 2009 [32]	3 times per week	Submaximal intensity HR 180 minus age	15–30 min	Endurance training: stationary cycling	
Klepin 2011 [33]	3 times per week, a total of 12 exercise sessions	Mild intensity for the walking phase	30–45 min Warm-up 5 min Walking phase up to 15min Strength and flexible program 15 min Second walking phase for up to 15 min Cool down for 5 min	Mixed modality Warm-up: walking in place and mild stretching Walking phase Strength and flexible program with resistance bands Second walking phase Cool down	
Pahl 2018 [8]	3 times per week	Moderate intensity RPE 14–16 (out of 20)	20 min Intervention: Each exercise lasted 30-60 sec. with 30-60 sec. rest between exercises and 60-120 sec. rest between sets	Intervention group: Whole body vibration: 3 sets of 2-4 different exercises on a sport vibration platform Control group: Aerobic exercise: stationary cycling	
Streckmann 2014 [34]	2 times per week	Moderate to high intensity Endurance training: Warm-up: 60–70% max HR Training: 70–80% max HR Strength training: maximum force	60 min Endurance training: 10–30 min Sensorimotor training: 11 min Strength training:4 min	Mixed modality: Warm-up: stationary cycling Aerobic training: treadmill or stationary cycling Sensorimotor training Strength training	Sensorimotor training: progressively increasing difficulty
Wehrle 2019 [35]	3 times per week	Light to moderate intensity Endurance group: 60–70% max HR and RPE 12–14 (out of 20) Resistance group: RPE 12–14 (out of 20)	30–45 min	Endurance group: stationary bicycle or a treadmill (continuous mode or if not possible interval to the method) Resistance group: bodyweight, small devices (dumbbells, elastic band), resistance machines, major muscle groups	Had to be adjusted daily, including the intensity, number of sets, and repetitions

### Safety

This section summarizes the studies with regard to the safety of the conducted intervention. Safety was measured by the occurrence or absence of adverse events. Neither was reported in five studies [27, 28, 30–32]. Four studies [8, 29, 33, 35] reported that no adverse events occurred. Streckmann et al. [34] reported that adverse events were neither statistically nor clinically meaningful. Only two studies [23, 24] mentioned the occurrence of adverse events using the National Cancer Institute: Cancer Therapy Evaluation Program Common Terminology Criteria version 3.0 and 4.0, respectively. These events were the observation of one supposed grade II musculoskeletal event in the first study [24] and four grade II musculoskeletal events of which three were unconfirmed and one probably related to the intervention in the other investigation [23].

### Feasibility

This section summarizes the studies with regard to the feasibility of the conducted intervention. Feasibility was reported in ten [8, 23, 24, 27, 30–35] of the twelve investigations. Of these, only four explicitly operationalized feasibility in their methods section. Alibhai et al. (2012) [24] evaluated feasibility through recruitment and retention rate. Alibhai et al. (2015) [23] determined recruitment, retention, and adherence rate. Furthermore, they expressed goals, which needed to be fulfilled to continue with a larger effectiveness trial. Klepin et al. [33] defined feasibility by recruitment rate, completion of exercise sessions, assessment completion, and barriers to recruitment. Pahl et al. [8] compared the training compliance of the two groups as an assessment for feasibility.

The recruitment rate was either directly reported in the respective study or could at least be calculated on the basis of other results in eight studies [8, 23, 24, 29, 30, 33–35] and varies from 28.6 [8] to 85.7% [30]. The recruitment period and recruitment time rate ranged from seven [8] to 39 [34] months and from 0.9 to 4.7 participants per month, respectively. The retention rate was reported or could be calculated in 11 studies [8, 23, 24, 27, 29–35] and varies from 52.4 [33] to 97% [24]. Since adherence and compliance were used interchangeably throughout the investigated studies, they were summarized in the term adherence. Adherence was reported in seven studies [8, 23, 24, 29, 33–35] and varies from 45.8 [24] to 80% [29]. Baumann et al. [28] and Klepin et al. [33] reported that an average of 2.4 (± 0.66) and 2.7 (±2.4) sessions per week were attended by the participants, respectively. Reasons why training interventions could not take place were reported by six studies [8, 23, 24, 29, 35] and are presented in Table 4. The most frequently named reason for no training participation was physical and psychological malaise, which was reported by all six studies [8, 23, 24, 27, 29, 33, 35].

**Table 4:** Reasons for no training participation

	Alibhai 12 (%) [24]	Alibhai 15 (%) [23]	Battaglini [27]	Bryant [29]	Klepin (%) [33]	Pahl (number of missed sessions) [8]	Wehrle [35]
Fatigue	33.5	23.3		reported			reported
Medical exclusion	14.9	1.7	reported			IG 9 CG 2	reported
Unavailable/sleeping/discharge	9.5	10.0			21.0	IG 13 CG 8	
Physical and psychological malaise	33.7	43.0	reported	reported	71.0	IG 19 CG 16	reported
Organizational difficulties	3.6	9.8				IG 1 CG 0	reported
Other	4.8	12.1			8.0		reported

The above table lists the reasons for why patients did not participate in a scheduled training session as reported in the included studies. The table shows either the reported share of a particular reason out of all missed training sessions in the respective study (Alibhai 12, Alibhai 15, Klepin) or shows the absolute count of how often a reason was mentioned for the intervention (IG) and control group (CG) (Pahl). Lastly, the table reflects if a reason for no participation was mentioned at least once without further quantification (Battaglini, Bryant, Wehrle).

Feedback from the participants on the exercise interventions was asked by four investigations [8, 29, 31, 33]. Overall, they reported that participants were pleased with the intervention and experienced it as beneficial.

Information about the adherence to other FITT criteria except frequency was not given by any study.

## Discussion

This systematic literature review presents an overview of the current literature regarding the feasibility and safety of exercise interventions in hematological patients during chemo- or induction therapy. All included studies claimed that their interventions were either feasible and/or safe. In most cases, a study was considered safe when there were no adverse events. It can be stated that there was no indication of long-lasting harm for any participants ascribable to the exercise intervention, independent of the chosen exercise type. However, it is important to mention that all investigations in total “only” included just over 200 participants, who received a variety of different exercise interventions. This is important to keep in mind because rarely occurring health risks might not have been observed simply due to the limited number of participants. For future investigations, it therefore remains important to keep the participants closely monitored to ensure patients’ safety. Furthermore, future studies still need to report any adverse events and/or safety issues as they occur.

The good to excellent retention rates in many studies suggest that, once recruited, the participants themselves experience beneficial effects and are highly motivated to keep active. Investigations asking participants for feedback, either through an interview or questionnaire, further support this, as they generally report satisfaction with the intervention. From these studies, it can be concluded that patients diagnosed with hematological malignance undergoing therapy, who agreed to participate in an exercise trial, are thankful for the opportunity of additional supportive therapy.

Nevertheless, the need for additional supportive therapy cannot be seen in the adherence. Adherence rates are an important indicator when investigating the feasibility of exercise interventions. The average adherence of those studies, which described adherence at all, was 64.3%. This rate is considerably lower than among other trials with patients diagnosed with cancer (70–85%) [39]. Moreover, it is not made transparent at all what exactly took place during a training session, although it is frequently reported that training sessions had to be adjusted due to the daily wellbeing of the participant, such as fatigue, fever, and more [23, 24, 27, 29, 31, 34, 35]. These adjustments might include changes in intensity, number of sets, number of repetitions, change in the type of exercise, and reduction in the time of the exercise/ session. However, a standardized, transparent summary of these adjustments is missing in all cases although it would be highly beneficial for future publications, not only to help constitutive investigations, but also to create a realistic depiction of the reality for practitioners. While the same information is often also missing in exercise intervention studies with other cancer patients [19–21, 40], it is even more important for the group under investigation here as safety-related side effects (like low platelet counts, fever, infections, low hemoglobin, etc.) that require adjustments of the training protocol are very common during this treatment. Furthermore, specific criteria, when and which specific adjustments should be made as well as guidelines on how to report these, are needed.

Although safety and feasibility were claimed in all publications, a closer examination of the methodological components of the included studies is needed to evaluate and contextualize the results in their respective study designs and aims and discuss whether these might impact the claimed safety and/or feasibility.

Examining the aims of the identified investigations lays open that they mostly follow different or no scientific definitions of a feasibility study. The CONSORT 2010 statement defines that randomized pilot and feasibility trials should primarily aim “to assess the feasibility of conducting the future definitive RCT” [9]. In other words, the paramount goal is to answering the questions of whether a larger RCT can and should be done and, if the answer is yes, how it should be done [9]. In most of the investigated studies, however, neither these questions nor a plan for a subsequent RCT was even mentioned (five out of twelve). In fact, the scope for their feasibility or pilot study is undefined in four out of twelve investigations.

Although limited-efficacy testing is certainly one appropriate area a feasibility study can focus on [10], the authors often do not explicitly indicate the limited nature of their study but might instead be overly optimistic with their conclusion on the effectiveness. However, this does not necessarily indicate an author’s scientific inability or even malicious intention. Instead, a more fundamentalistic critique towards the scientific publishing system might be appropriate. The author’s might feel pressured towards overly optimistic interpretations of their results as some scientific journals do not accept feasibility studies. More generally, since studies on publication biases show that it is more likely to publish a study if it demonstrates beneficial effects [36, 37], researchers might feel a constrain to over-interpretation or extending pilot and feasibility studies beyond their defined scope [38]. Therefore, it is important to consider that the available data researchers can find in databases might be incomplete if investigations with an assumed less “scientific” relevance did not make it to the publication stage.

Lastly, pilot and feasibility studies are trials with a limited sample size by design. Although it comes with additional resource burdens, it remains desirable that future studies are executed with larger sample sizes for multiple reasons, e.g., to investigate undesirable side effects better [8, 27]. However, it turns out that low recruitment rates already seem to be a problem even in these small investigations. Problems with recruitment went as far as some trials needing to be stopped before reaching the aimed number of participants. Bryant et al. [29] point out that it might be due to the strict eligibility criteria, with regard to a cardio-pulmonary exercise testing, but with only two trials reaching a recruitment time rate over three participants per month, this seems to be a common problem which might benefit from further investigation. Another downside of larger studies that needs to be considered is that larger studies are not only more costly, but since they usually take more time, the enlarged timespan also increases the chance of a change in medical therapy during the investigation, which might contradict the inclusion criteria.

Besides the main focus of this systematic review of answering whether or not exercise during treatment is feasible and/or safe, two important key takeaways were drawn from analyzing the investigated studies. The first one addresses both the logistics when carrying out the exercise sessions and safety of the patient [24]. It is important to recall that exercise sessions can also be carried out in a hospital room with limited space and limited equipment [33]. The complexity and difficulty of implementing exercise interventions do not only come from the in-hospital setting but additionally that patients sometimes are not allowed to leave their rooms due to safety concerns. Therefore, it cannot be stressed enough that the hygiene of the used equipment is very important, due to a higher vulnerability to infections and the patients’ therapy-weakened immune systems. Additionally, the presence of chest or arm ports or even intravenous lines and poles, which cannot be disconnected during training, demand a well-planned and also flexible exercise protocol to be adjustable for the individual situation [24, 27].

The second insight promises to enhance the outcome quality. Outcome quality might be increased by adding strategies found in health psychology like cognitive-behavioral tools and self-monitoring of exercise [23], as it might lead to an improvement in adherence numbers. Additionally, the involvement and education of patients and family members in the program and about potential benefits might also enhance motivation [23, 33].

While this study brought to light that exercise appears to be feasible and safe for the population under investigation, it also faces three limitations. First, the title and abstract screening were only conducted by one author, which might have introduced unintended consequences. In future revisions of this review, a second author should participate in the initial screening. Second, no quality assessment was conducted. Future investigations might want to find a way to compare and assess the quality between studies of different types (RCT vs feasibility study vs pilot study). Lastly, this review could have been even more focused on AML patients only. Instead, similar diseases were included, which lead to a richer foundation for the review but may lead to less precise results.

In this systematic review, we summarized the literature regarding the feasibility of exercise training in inpatient patients diagnosed with hematological cancer until April 2022. Across all publications, researchers document the safety and feasibility of exercise interventions. Nevertheless, recruitment rates need improvements to succeed in higher numbers of participants. Additionally, a higher recruitment rate might ultimately reduce the overall costs of a feasibility or pilot study due to time savings when a target sample size is met earlier. One possible option, which appears to be rarely exerted, might be to implement multi-centered approaches. Lastly, to increase the overall quality of studies and help evaluate the effectivity of exercise programs and optimize the exercise regimes, the authors should report methodological details for both the planned and executed investigations more elaborately.

## Supplementary information

ESM 1
