# Supplementary material for: Safety and feasibility of exercise interventions in patients with hematological cancer undergoing chemotherapy: a systematic review
Source: Support Care Cancer. 2023 May 15;31(6):335. doi: 10.1007/s00520-023-07773-9 (PMC10183429; doi:10.1007/s00520-023-07773-9)
Supplement: Supplementary file 1 — ESM 1 [file 520_2023_7773_MOESM1_ESM.docx]

**Safety and feasibility of exercise interventions in patients with hematological cancer undergoing chemotherapy: A systematic review**

**Abstract**

Objective: Exercise during and after cancer treatment has established quality of life and health benefits. However, particularly for patients with hematological cancer clear recommendations regarding the safety and feasibility for exercise are under-investigated. The aim of our systematic review was to summarize the literature regarding feasibility and safety of exercise interventions in patients diagnosed with hematological cancer undergoing chemotherapy.

Method: A systematic literature review was conducted using the PubMed, SPORTDiscus, MEDLINE, science direct and web of science electronic databases. Eligible studies were scientific publications reporting the feasibility and/or safety of an exercise intervention program carried out in inpatient patients diagnosed with hematological cancer undergoing chemotherapy.

Result: Out of 12 studies (six RCTs) included in this review, six investigations reported results with regards to safety and 10 with regards to feasibility. While all studies claim that their exercise interventions were safe and/or feasible, it is noteworthy that this claim often remains unsupported as detailed information on how feasibility of the intervention was asserted is missing.

Conclusion: Exercise appears to be safe and feasible in hematological cancer patients. However, due to a striking lack of information on how feasibility of the intervention was asserted, contextualizing the results and deducing recommendations for further studies remains challenging. Further research should therefore incorporate information on the execution of the exercise intervention in more detail.

Keywords: Leukemia, lymphoma, physical activity, feasibility, safety

**Introduction**

Hematological malignances, such as acute leukemia and aggressive lymphoma, are life-threatening and fast progressing diseases which cause symptoms like anemia, vulnerability to infections, fever, bleeding, nausea, weight loss, and fatigue^1^. A curative treatment requires a rapid hospitalization, initial high dose chemo- or induction therapies, and comes along with multiple toxicities and extended bedrest^2,3^. The symptoms caused by the disease, side effects of the treatment (e.g. cytopenia, depression, fatigue), and inactivity can lead to physical and mental deconditioning, which causes decline in quality of life^4,5^.

A variety of exercise intervention studies have demonstrated a beneficial effect on both physical and psychological outcomes, even stating the need for implementation of different exercise regimes into usual care in cancer patients^6–8^. This suggests that exercise might be as promising in patients diagnosed with acute leukemia or aggressive lymphoma as it is for patients diagnosed with breast, colorectum, or prostate cancer. Nevertheless, findings for solid tumors might not be applicable to systemic tumors. Furthermore, variations in systemic tumors need to be considered. Conclusions from solid tumors might apply differently to patients diagnosed with acute leukemia compared to those diagnosed with chronic leukemia since these patients underlie unequal prerequisites due to differences in the aggressiveness of their specific treatment (e.g., higher treatment related mortality often caused by infections requiring high hygiene standards during training, extended bed-rest, lower blood counts, and multiple toxicities leading to physical deconditioning for patients with acute leukemia).

Proving the beneficial effect of exercise interventions, requires studies, which are specifically designed for hospitalized hematological patients under treatment.

The gold standard to test the effectiveness of an exercise intervention is a randomized controlled trial (RCT), which are often not only expensive but also organizationally demanding. To minimize the cost and maximize the success, it is essential to rule out any unexpected complications as best as possible prior to the start of the RCT. Feasibility studies are a useful method to test the RCT’s processes, as they question ‘whether a future trial can be done, should be done and if so, how’^9^ before embarking on the actual RCT. While testing the RCT’s processes a feasibility study can focus on a wide array of different areas such as acceptability, demand, implementation, practicality, adaptation, integration, expansion and limited-efficacy testing^10^. Hence, feasibility studies can become complex studies themselves and therefore rarely cover all the potential disturbing factors simultaneously. Feasibility studies must not to be confused with pilot studies, which are considered a subset of feasibility studies, but follow the study protocol of the future RCT on a smaller scale^9,11^ instead of focusing on testing the RCT’s processes.

To the knowledge of the authors, a variety of researchers already investigated the potential of exercise interventions in hematological patients. Nevertheless participants were not necessarily diagnosed with such severe types as investigated in this study. Additionally, participants were in different stages of their therapy and the focus of these trials was mostly on the effectivity on the training program, not giving clear recommendations or suggestions with regards to the feasibility and challenges of the chosen exercise program^12–14^.

To contribute to improving the scientific standard of future intervention studies this paper aims to systematically review the current literature on feasibility studies of exercise and physical activity interventions in patients diagnosed with hematological malignances. Additionally, it further investigates the study designs and aims of the included investigations and evaluates which influence they might have on the feasibility and safety assessment of the target group.

**Method**

This systematic literature review was conducted in accordance with the PRISMA (Preferred Reporting Items for Systematic Reviews and Meta-Analyses) guidelines^15^.

**Eligibility criteria**

Only articles published in peer-reviewed scientific journals with full-text access in English language were included. Further in- and exclusion criteria were defined using the PICOS (Population, Intervention, Comparison, Outcome, Study Design) scheme^16^. Population: The participants of the included studies needed to be older than 18 years and be currently diagnosed with acute leukemia or an aggressive form of lymphoma. Regardless of entity the participants needed to be hospitalized to undergo induction or high-dose chemotherapy. Intervention: Studies were eligible for this review if they included any kind of exercise or physical activity intervention, which was scheduled next to the induction/high-dose chemotherapy. Studies were excluded if they primarily investigated behavioral interventions, or the exercise intervention was not supervised and only advised. Comparison: As this investigation focused primarily on the feasibility and safety of exercise interventions, a control group was less relevant. Outcome: Any reporting of feasibility was evaluated, with using the word feasibility or feasible being sufficient to be included in this review. Additionally, adherence, retention and recruitment rates which are often related to feasibility, were analyzed. Study design: To get the broadest view on the existing literature, randomized controlled trials were included alongside non-randomized controlled trials, and uncontrolled trials.

**Search strategy**

This systematic literature review was conducted searching the following electronic databases for relevant literature: PubMed, SPORTDiscus, MEDLINE via EBSCOhost, science direct and web of science. The search was completed by AG on April 10, 2022 and was not limited to an earliest publishing year. The detailed search strings are provided in Table 1.

Title and abstracts of all articles were screened according to the eligibility criteria by AG. The remaining full text articles were subsequently reviewed for eligibility by AG and KG. Relevant information of the final publications was extracted independently by AG and KG and disagreements were solved through discourse.

Table 1 Search string for each database

| Databases | Search strategy |
| --- | --- |
| PubMed | (((leukem*[Title/Abstract] OR leukaem*[Title/Abstract] OR lymph*[Title/Abstract] OR hematological[Title/Abstract]) AND (chemotherapy* OR induction therap*)) AND (exercise[Title/Abstract] OR training[Title/Abstract] OR sport*[Title/Abstract] OR resistance[Title/Abstract] OR aerobic[Title/Abstract] OR strength[Title/Abstract] OR walking[Title/Abstract] OR ergometer[Title/Abstract] OR physiotherap*[Title/Abstract] OR physical therap*[Title/Abstract] OR endurance[Title/Abstract] OR yoga[Title/Abstract] OR tai chi[Title/Abstract])) AND (feasib* OR attendance OR adherence OR retention OR recruitment OR pilot OR safety OR adverse events OR adverse effects) |
| SPORTDiscus | ((leukem* OR leukaem* OR lymph* OR hematological) AB abstract) AND ((chemotherapy* OR induction therap*) TX All Text) AND ((exercise OR training OR sport* OR resistance OR aerobic OR strength OR walking OR ergometer OR physiotherapy* OR physical therap* OR endurance OR physical activity) AB abstract) AND feasib* OR attendance OR adherence OR retention OR recruitment OR pilot OR safety OR adverse events OR adverse effects) TX All Text) |
| MEDLINE | ((leukem* OR leukaem* OR lymph* OR hematological) AB abstract) AND ((chemotherapy* OR induction therap*) TX All Text) AND ((exercise OR training OR sport* OR resistance OR aerobic OR strength OR walking OR ergometer OR physiotherapy* OR physical therap* OR endurance OR physical activity) AB abstract) AND feasib* OR attendance OR adherence OR retention OR recruitment OR pilot OR safety OR adverse events OR adverse effects) TX All Text) |
| Science direct | (Article (feasibility OR adherence OR pilot OR feasible OR safety) AND (chemotherapy OR induction therapy)) AND (Title, abstract or author-specified keywords (leukemia OR leukaemia OR lymphoma OR hematological) AND (exercise OR training OR sport OR physical activity OR resistance)) |
| Web of science | (leukem* OR leukaem* OR lymph* or hemtological (Abstract)) AND (chemotherap* OR induction therap* (All Fields)) AND exercise OR training OR sport* OR resistance OR aerobic OR strength OR walking OR ergometer OR physiotherap* OR physical therap* OR endurance OR physical activity (Abstract)) AND (feasib* OR attendance OR adherence OR retention OR recruitment OR pilot (All fields)) |

**Data extraction**

Interestingly, definitions of ‘feasibility’ and ‘pilot study’ are not as distinct as expected. Consequently, there is a large variety in possible study designs for investigations testing feasibility. To structure the search results, studies are divided into randomized pilot studies, non-randomized pilot studies, and other feasibility studies as suggested by Eldridge et al.^17^. To give a detailed and structured view into the different exercise interventions, they are arranged according to the FITT (frequency, intensity, time and type of exercise) principles as this is a common procedure to describe exercise interventions in depth^18–22^. Although safety is also considered under feasibility aspects occasionally, it is described separately in this review to emphasize the importance of safe exercise interventions for an already vulnerable population. Both feasibility and safety are reported as in the investigated studies. For safety, the mere absence of adverse events suffices to consider a study safe. For feasibility additionally, recruitment rate, recruitment period, recruitment time rate, retention rate, and training participation were investigated. The recruitment rate was defined by number of participants who signed the informed consent divided by number of eligible patients who were screened^23,24^.The recruitment period represents the time (in month) between first and last recruitment. As described by Walters et al.^25^ the first and last month were counted as whole if not described differently. The recruitment time rate was calculated by the number of recruited participants per month^25^. Finally, the retention rate was determined by the number of participants attending the final assessment divided by participants randomized^26^. Finally, training participation was presented as adherence (attended exercise sessions divided by planned exercise sessions) or as training session per week.

Figure 1 Flow diagram for literature search of the systematic review

**Identification of studies via databases and registers**

Records removed *before screening*:

Duplicate records removed (n =1570 )

Records marked as ineligible by automation tools (n = 0)

Records removed for other reasons (n = 0)

Records identified from:

Medline (n=1137)

PubMed (n=1513)

Web of Science (N=1083)

SportDiscus (n=58)

Science direct (n= 896)

Other sources (n=0)

**Identification**

Records screened

(n = 3117)

Records excluded (no automation tools were used)

(n = 3095)

Reports sought for retrieval

(n = 22)

Reports not retrieved

(n = 0)

**Screening**

Reports assessed for eligibility

(n = 22)

Reports excluded:

Not inpatient (n = 7)

No chemo- /induction therapy (n = 1)

Mixed group patients, no separate analysis (n = 1)

Not supervised (n = 1)

Studies included in review

(n = 12)

Reports of included studies

(n = 12)

**Included**

**Results**

The literature search executed on electronic databases yielded 4,687 results, of which 3,117 remained after the removal of duplicates. Of these, 12 met the inclusion criteria and were included in this systematic literature review. The full identification, screening, and inclusion process is presented in Figure 1. All included studies consider themselves ‘pilot-studies’ or claim any kind of researched feasibility of their chosen activity intervention^8,23,24,27–35^, even if feasibility is neither a declared primary nor secondary outcome in some studies. The group of patients varies between patients diagnosed with acute myeloid leukemia (AML) only, acute leukemia, lymphoma only, and a mixed group of leukemia and lymphoma patients, which were investigated in five^23,24,27,30,33^, two^29,35^, one^34^ and four studies^8,28,31,32^, respectively. A more detailed overview of the study characteristics and interventions are presented in Table 2.

**Study Designs and Aims**

Six of the included studies followed a randomized controlled study design^8,23,29,30,34,35^, four had no control group^24,27,32,33^, one was a matched pair control^28^, and one had a convenience sample^31^ (patients, who chose to not participate in accordance with the exercise protocol, were asked to be part of the control group). It was further possible to classify all included studies into one of the following groups: randomized pilot studies^8,29,35^, non-randomized pilot studies^24,28,32,33^, or feasibility studies^23,27,30,31,34^. This classification was done in accordance with the conceptual framework to define feasibility and pilot studies of Eldridge et al. ^17^. In a first step, the following sections will elaborate in more detail on the study design and aims of each of the studies per each classified group to contextualize one study’s results with the results from studies with a similar study design.

**Randomized pilot studies**

Bryant et al. ^29^ performed a two-armed investigation with an intervention (mixed-modality; n=9) and a control (usual care, n=9) group. The study aims were to (i & ii) examine the effects of the intervention on a variety of physical and psychological health outcomes, and (iii) evaluate the effect of intervention on adherence to exercise. Pahl et al. ^8^ conducted a two-armed investigation with an intervention (whole body vibration (WBV), n=6) and an active control (cycling, n=5) group, to evaluate the following study aims: (i) feasibility of WBV by investigating training compliance, exercise-related adverse events, and a self-designed questionnaire; (ii) effects on functional performance and mobility. Wehrle et al. ^35^ performed a three-armed trial with an endurance (n=9), a resistance (n=10), and a control (n=10) group. The study aim of this investigation was to investigate independent effects of the two interventions on physical capacity and QOL.

**Non-randomized pilot studies**

Out of the four non-randomized pilot studies, three^24,32,33^ were designed without a control group. Alibahai et al.^24^ (n= 35) aimed to (i) determine recruitment, retention, and ability to participate in the intervention, (ii) provide efficacy estimates on physical fitness outcome measures, (iii) examine the safety of the program, (iv) provide estimates on the effects of the intervention on QOL and fatigue, and (v) understand the impact of exercise on AML treatment tolerability. Elter et al.^32^ (n=12) addressed the feasibility of the intervention as well as investigating any effect. Klepin et al. ^33^ (n=24) assessed (i) the feasibility of the intervention by investigating the recruitment rate, number of exercise sessions, percentage of participants completing assessments, and barriers to recruitment, as well as (ii) preliminary data on the efficacy of the training on different health outcomes. The fourth non-randomized pilot study conducted by Bauman et al.^28^, following a matched-pair design, aimed to investigate (i) the effect of training interventions on the incidence of fever and pneumonias, (ii) changes in neutrophile- or leukocyte counts, and (iii) time spent in the hospital.

**Feasibility studies**

Three studies^23,30,34^ were identified as feasibility studies following a randomized controlled trial design. The phase II randomized controlled feasibility trial by Alibhai et al.^23^ aimed to determine (i) the feasibility (via rates of recruitment, retention and adherence), safety (by reporting adverse events) and preliminary evidence of efficacy of the training program on health related outcomes, and (ii) the impact of the intervention on treatment tolerability by investigating the length of stay, development of sepsis, intensive care unit admissions, and delays in further treatment. In their randomized controlled trial Chang et al.^30^ aimed to develop and preliminarily examine the effects of a clinical feasible exercise intervention. Streckmann et al.^34^ evaluated the effect of the training intervention on (i) QOL, (ii) peripheral neuropathy, (iii) activity levels, (iv) balance control on static surfaces, and (v) balance control on dynamic surfaces.

A two armed trial with convenience sample was executed by Duregon et al.^31^. They investigated (i) the feasibility of the exercise program, (ii) the implementation of the support of tablets in the exercise routine, and (iii) the effects of the training program on physical function. Battaglini et al.^27^ examined in their one-armed study (i) the feasibility of the intervention, and (ii) the effect of the intervention on health-related outcomes.

**Study Interventions by FITT-criteria**

This section summarizes the studies with regards to the FITT criteria to give an overview of the conducted training interventions. A mixed modality exercise program was investigated by seven studies^23,24,27,29,31,33,34^, three investigated an endurance exercise program^28,30,32^, one study compared whole body vibration against endurance training^8^, and one study investigated endurance as well as resistance exercises in a three-armed trial^35^. The duration of the interventions varied between two to four weeks^8^ and ranged up to 36 weeks^34^. Training frequencies ranged between two^34^ and five^30,31^ times per week with a duration between 12 minutes^30^ and 60 minutes^23,34^. A more detailed overview of the frequencies, intensity, time, and type of exercise is presented in Table 3.

**Safety**

This section summarizes the studies with regards to the safety of the conducted intervention. Safety was measured by the occurrence or absence of adverse events. Neither was reported in five studies^27,28,30–32^. Four studies^8,29,33,35^ reported that no adverse events occurred. Streckmann et al. ^34^ reported that adverse events were neither statistically nor clinically meaningful. Only two studies^23,24^ mentioned the occurrence of adverse events using the National Cancer Institute: Cancer Therapy Evaluation Program Common Terminology Criteria version 3.0 and 4.0, respectively. These events were the observation of one supposed grade II musculoskeletal event in the first study^24^ and four grade II musculoskeletal events of which three were unconfirmed and one probably related to the intervention in the other investigation^23^.

Table 2 Overview study characteristics

| Author | Study type | Diagnosis & Treatment | Age (years) | Intervention | Control | Adverse events | Recruitment rate, recruitment period, recruitment time rate | Retention rate | Training participation (Adherence % or Training per week) | Comments |
| --- | --- | --- | --- | --- | --- | --- | --- | --- | --- | --- |
| Alibhai 2012 ^24^ | Non-randomized pilot study | Newly diagnosed AML or relapsed AML | 56.4 (±12.9) | Individualized, supervised, mixed-modality exercise program (n=35) | No control group per design | One possible grade II musculoskeletal event occurred | 67%; 9 months; 3.9 participants/ month | 97% | 45.8% |  |
| Alibhai 2015^23^ | Feasibility study | Newly diagnosed AML or relapsed AML | IG: 58 (±13.9)  CG: 52 (15.8) | Individualized, supervised, mixed-modality exercise program, 4-6 weeks  (n=57) | Usual care (n=24) | 4 grade II musculoskeletal events (3 possible and 1 probably related to the intervention) | 56%; 21 months; 3.9 participants/ month | 96% | 54% |  |
| Battaglini 2009^27^ | Feasibility study | Newly diagnosed AML or relapsed AML | 35.7 (± 8.9) | Individualized prescriptive aerobic exercise program, 3-5 weeks (n=10)  Mixed modality | No control group per design | Not reported | Not reported; not reported | 80% | Not reported | Participants were excluded if they were older than 55 years |
| Baumann 2012^28^ | Non-randomized pilot study | Both groups:  AML: 7  ALL: 2  HL: 3  NHL: 6 | IG:  46.11 (±16.22)  CG:  45.22 (±15.21) | Three weeks ergometer training (n=18) | Usual care (n=18) | Not reported | Missing information; 17 months; 2.1 participant/ month | Not reported | Training per week: 2.4 (± 0.66) times, 25 (±7.9) minutes |  |
| Bryant 2018^29^ | Randomized pilot study | IG:  ALL: 1  AML: 7  CG:  ALL: 1  AML: 8 | IG: 52 (± 13)  CG: 49 (±15) | Individualized, supervised, mixed-modality exercise program, (n=9) | Usual care (n=9) | No adverse events occurred | 48.6%; 14 months; 1.3 participants/ month | 94.4% | 80% adherence of visits completed, with a mean of 6 sessions attended per week | Initial recruitment plan was for 30 patients, close early due to recruitment issues – that is, identifying patients without comorbidities |
| Chang 2008^30^ | Feasibility study | AML | IG: 49.4 (±15.3)  CG: 53.3 (±13.6) | Supervised walking exercise program, 3 weeks (n=11) | 5x/week non-invasive routine care by a research assistant, (n=11) | Not reported | 85.7%; not reported | 91.7% | Not reported |  |
| Duregon 2019^31^ | Feasibility study | IG:  AML:15  ALL:0  NHL: 5  HL: 7  MM: 3  CG:  AML: 5  ALL:1  NHL:3  HL:2  MM:1 | IG: 48.7 (±14.4)  CG: 50.8 (±12.7) | Individualized, supervised mixed-modality exercise program (n=30) | Usual care (n=12) | Not reported | Not reported; 9 months; 4.7 participants/ month | 59.5% | Not reported |  |
| Elter 2009^32^ | Non-randomized pilot study | AML: 7  ALL: 1  NHL: 4 | 44.16 (±13.85) | Ergometer training (n=12) | No control group per design/ n.a. | Not reported | Not reported | 66,67% | Not reported |  |
| Klepin 2011^33^ | Non-randomized pilot study | AML | 65.1 (±7.8) | Mixed-modality exercise program (n= 21) | No control group per design/ n.a. | No adverse events occurred | 43.6%; 19 months; 1.3 participants/ month | 52.4% | 2.7 per week (range 0-8, ± 2.4), | Inclusion criteria: Age ≥ 50 years |
| Pahl 2018^8^ | Randomized pilot study | IG:  AML: 1  ALL: 1  APL:1  NHL: 2  t-cell L: 1  CG:  AML:4  MM:1 | IG: 47 (19-62)  CG: 56 (32-63) | Supervised whole body vibration (n=10) | Supervised aerobic exercise  (n=10) | No adverse events occurred | 28.6%; 7 months; 2.9 participants/ month | 55% | IG 62%;  CG 67% |  |
| Streckmann 2014^34^ | Feasibility study | Lymphoma patients | IG: 44 (20-67)  CG: 48 (19-73) | 36 weeks, supervised mixed-modality exercise program (n=30) | Usual care (n=31) | No adverse events are statistically and clinically meaningful | 32.8%; 39 months; 1.6 participants/ month | 83.6% | 65%  (Highest for SMT, lowest for strength) | low recruitment-rate lead to end of study after three years not reaching the goal of 92 participants per group |
| Wehrle 2019^35^ | Randomized pilot study | AL | IGa: 47.7 (21.9-63.4)  IGb: 47.4 (41.2-62.2)  CG:  50.6 (35.0-58.1) | Endurance training  (IGa n=9)  Resistance training (IGb: n=10) | 3x/week low intensity mobilization and stretching program  (n=10) | No adverse events occurred | 74.4% (1); 33 months; 0.9 participants/ month | 75.9% | IGa: 68.9%  IGb: 76.0%  CG: 60.0% | Low recruitment, recruitment process was stopped after 2,5 years |

Abbreviations: IG: Intervention group; CG: Control group; AML: Acute Myeloid Leukemia; ALL: Acute Lymphoblastic Leukemia; HL: Hodgkin Lymphoma; NHL: Non-Hodgkin Lymphoma; MM: Multiple Myeloma; t-cell L: t-cell Lymphoma; (1) based on calculation; deviates from the publicized number by ca. 10%

Table 3 FITT criteria of study interventions

| Author | Frequency | Intensity | Time | Type of exercise | Comments |
| --- | --- | --- | --- | --- | --- |
| Alibhai 2012^24^ | 4-5 times per week | Light to moderate intensity  Aerobic exercise: RPE 3-6 (out of 10) or 50-75% of their heart rate reserve (HRR) | 30-45 min.  Aerobic: 10-40 min.  Resistance 10-25 min.  Flexible training: 5-10 min. | Mixed modality  Aerobic exercise: walking or stationary cycling  Resistance training: resistance bands and/or free weights, major muscle groups  Flexible training: static stretching | HRR was found to be obstructive for patients due to intravenous lines 🡪 was dropped  Progression and adaptation:  Aerobic: first increase in length then in intensity  Resistance: increasing number of sets, repetitions, or resistance |
| Alibhai 2015^23^ | 4-5 times per week | Light to moderate intensity  Aerobic exercise: RPE 3-6 (out of 10) AND 50-75% of their heart rate reserve | 30-60 min. | Mixed modality  Aerobic exercise: treadmill, hall walking or stationary cycling  Resistance training: body weight, resistance bands and/or free weights, major muscle groups  Flexible training: static stretching | Time: depending on participant ability and clinical symptoms  Progression and adaptation:  Aerobic: first increase in length then in intensity  Resistance: increasing number of sets, repetitions, or resistance |
| Battaglini 2009^27^ | 3-4 times per week  (min. 36h rest between sessions) | Submaximal intensity  Endurance: 40-50% HRR and RPE < 5 (out of 10)  Resistance exercise: < 5 (out of 10) | 30 min. each bout  Light stretching: 3-5 min.  Endurance training: 5-10 min.  Resistance training: 5-15 min.  Core exercise: 5-10 min. | Mixed modality  Light stretching  Endurance training: treadmill or stationary cycling  Resistance training: dumbbells, resistance bands, and exercise ball  Core exercise | Time: depended on patient’s physical state on the day  Each session was divided into two bouts, one in the morning and one in the afternoon |
| Baumann 2012^28^ | 3 times per week | Moderate intensity  RPE 13 (out of 20); max. HR 180-age; 80% max. HR | 20-30 min. | Endurance exercise: stationary cycling |  |
| Bryant 2018^29^ | 4 times a week, | Light to moderate intensity:  Aerobic 50-70% HRR  Resistance intensity increased using a 10 Rep. Max raining protocol | 20-40 min.  Aerobic training: 5-15 min.  Resistance training: 10-20 min.  Stretching 5 min. | Mixed modality  Aerobic: walking or stationary cycling  Resistance: resistance bands  Stretching | Was adapted based on the patient’s physical limitations  Twice a day |
| Chang 2008^30^ | 5 times per week | Light intensity  Target heart rate: resting heart rate plus 30 | 12 min. | Hallway walk |  |
| Duregon 2019^31^ | 5 times per week | Light to moderate intensity  RPE: 11-13 (out of 20) | 15-30 min. | Mixed modality  Warm up: breathing exercise and joint mobility  Central part: proprioception, resistance (1-2 sets, with 8-101 repetitions and 12 min rest) and flexible training  Cool down: Relaxing and breathing | Additionally, workout autonomously, exercise program on tablet)  Performance and exercise volume could vary everyday, based on the health conditions of patients  During aplasia no strength training |
| Elter 2009^32^ | 3 times per week | Submaximal intensity  HR 180 minus age | 15-30 min. | Endurance training: stationary cycling |  |
| Klepin 2011^33^ | 3 times per week, a total of 12 exercise sessions | Mild intensity for  walking phase | 30-45 min.  Warm up 5 min.  Walking phase up to 15min.  Strength and flexible program 15 min.  Second walking phase up to 15 min.  Cool down 5 min. | Mixed modality  Warm up: walking in place and mild stretching  Walking phase  Strength and flexible program with resistance bands  Second walking phase  Cool down |  |
| Pahl 2018^8^ | 3 times per week | Moderate intensity  RPE 14-16 (out of 20) | 20 min  Intervention:  Each exercise lasted 30-60 sec. with 30-60 sec. rest between exercises and 60-120 sec. rest between sets | Intervention group:  Whole body vibration:  3 sets of 2-4 different exercises on a sport vibration platform  Control group:  Aerobic exercise: stationary cycling |  |
| Streckmann 2014^34^ | 2 times per week | Moderate to high intensity  Endurance training:  Warm-up: 60-70% max HR  Training: 70-80% max HR  Strength training: maximum force | 60 min.  Endurance training: 10-30 min.  Sensorimotor training: 11 min.  Strength training:4 min. | Mixed modality:  Warm up: stationary cycling  Aerobic training: treadmill or stationary cycling  Sensorimotor training  Strength training | Sensorimotor training: progressively increasing difficulty |
| Wehrle 2019^35^ | 3 times per week | Light to moderate intensity  Endurance group:  60-70% max HR and RPE 12-14 (out of 20)  Resistance group:  RPE 12-14 (out of 20) | 30-45 min. | Endurance group:  stationary bicycle or a treadmill  (Continuous mode or if not possible interval to method)  Resistance group: bodyweight, small devices (dumbbells, elastic band), resistance machines, major muscle groups | Had to be adjusted daily, including the intensity, number of sets, and repetitions |

**Feasibility**

This section summarizes the studies with regards to the feasibility of the conducted intervention. Feasibility was reported in ten^8,23,24,27,30–35^ of the twelve investigations. Of these, only four explicitly operationalized feasibility in their methods section. Alibhai et al. (2012)^24^ evaluated feasibility through recruitment and retention rate. Alibhai et al.(2015)^23^ determined recruitment, retention and adherence rate. Furthermore, they expressed goals, which needed to be fulfilled to continue with a larger effectiveness trial. Klepin et al.^33^ defined feasibility by recruitment rate, completion of exercise sessions, assessment completion, and barriers to recruitment. Pahl et al.^8^ compared the training compliance of the two groups as assessment for feasibility.

Recruitment rate was either directly reported in the respective study or could at least be calculated on the basis of other results in eight studies^8,23,24,29,30,33–35^ and varies from 28.6%^8^ to 85.7%^30^. The recruitment period and recruitment time rate ranged from 7^8^ to 39^34^ months and from 0.9 to 4.7 participants per month, respectively. Retention rate was reported or could be calculated in 11 studies^8,23,24,27,29–35^ and varies from 52.4%^33^ to 97%^24^. Since adherence and compliance were used interchangeably throughout the investigated studies, they were summarized in the term adherence. Adherence was reported in seven studies^8,23,24,29,33–35^ and varies from 45.8%^24^ to 80%^29^. Baumann et al.^28^ and Klepin et al.^33^ reported that an average of 2.4 (± 0.66) and 2.7 (±2.4) sessions per week were attended by the participants, respectively. Reasons why training interventions could not take place were reported by six studies^8,23,24,29,35^ and are presented in Table 4. The most frequently named reason for no training participation was physical and psychological malaise, which was reported by all six studies^8,23,24,27,29,33,35^.

Feedback of the participants on the exercise interventions was asked by four investigations^8,29,31,33^. Overall, they reported that participants were pleased with the intervention and experienced it as beneficial.

Information about the adherence of other FITT criteria except frequency was not given by any study.

*Table 4 Reasons for no training participation*

|  | Alibhai 12 (%)^24^ | Alibhai 15 (%) ^23^ | Battaglini^27^ | Bryant^29^ | Klepin (%) ^33^ | Pahl (number of missed sessions)^8^ | Wehrle^35^ |
| --- | --- | --- | --- | --- | --- | --- | --- |
| Fatigue | 33.5 | 23.3 |  | reported |  |  | reported |
| Medical exclusion | 14.9 | 1.7 | reported |  |  | IG 9  CG 2 | reported |
| Unavailable/  sleeping/ discharge | 9.5 | 10.0 |  |  | 21.0 | IG 13  CG 8 |  |
| Physical and  psychological malaise | 33.7 | 43.0 | reported | reported | 71.0 | IG 19  CG 16 | reported |
| Organizational  difficulties | 3.6 | 9.8 |  |  |  | IG 1  CG 0 | reported |
| Other | 4.8 | 12.1 |  |  | 8.0 |  | reported |

The above table lists the reasons for why patients did not participate in a scheduled training session as reported in the included studies. The table shows either the reported share of a particular reason out of all missed training sessions in the respective study (Alibhai 12, Alibhai 15, Klepin), or shows the absolute count of how often a reason was mentioned for the intervention (IG) and control group (CG) (Pahl). Lastly, the table reflects if a reason for no participation was mentioned at least once without further quantification (Battaglini, Bryant, Wehrle).

**Discussion**

This systematic literature review presents an overview of the current literature regarding the feasibility and safety of exercise interventions in hematological patients during chemo- or induction therapy. All included studies claimed that their interventions were either feasible and/or safe. In most cases a study was considered safe when there were no adverse events. It can be stated that there was no indication of long-lasting harm for any participants ascribable to the exercise intervention, independent of the chosen exercise type. However, it is important to mention that all investigations in total ‘only’ included just over 200 participants, who received a variety of different exercise interventions. This is important to keep in mind because rarely occurring health risks might not have been observed simply due to the limited number of participants. For future investigations it therefore remains important to keep the participants closely monitored to ensure patients’ safety. Furthermore, future studies still need to report any adverse event and/or safety issue as they occur.

The good to excellent retention rates in many studies suggest that, once recruited, the participants themselves experience beneficial effects and are highly motivated to keep active. Investigations asking participants for feedback, either through an interview or questionnaire, further support this, as they generally report satisfaction with the intervention. From these studies it can be concluded that patients diagnosed with hematological malignance undergoing therapy, who agreed to participate in an exercise trial, are thankful for the opportunity of additional supportive therapy.

Nevertheless, the need for additional supportive therapy cannot be seen in the adherence. Adherence rates are an important indicator when investigating the feasibility of exercise interventions. The average adherence of those studies, which described adherence at all, was 64.3%. This rate is considerably lower than among other trials with patients diagnosed with cancer (70-85%)^39^. Moreover, it is not made transparent at all what exactly took place during a training session, although it is frequently reported that training sessions had to be adjusted due to the daily wellbeing of the participant, such as fatigue, fever, and more^23,24,27,29,31,34,35^. These adjustments might include changes in intensity, number of sets, number of repetitions, change in type of exercise, and reduction in the time of the exercise/ session. However, a standardized, transparent summary of these adjustments is missing in all cases although it would be highly beneficial for future publications, not only to help constitutive investigations, but also to create a realistic depiction of the reality for practitioners. While the same information is often also missing in exercise intervention studies with other cancer patients^19–21,40^ it is even more important for the group under investigation here as safety related side effects (like low platelet counts, fever, infections, low hemoglobin etc.) that require adjustments of the training protocol are very common during this treatment. Furthermore, specific criteria, when and which specific adjustment should be made as well as guidelines on how to report these, are needed.

Although safety and feasibility were claimed in all publications, a closer examination of the methodological components of the included studies is needed to evaluate and contextualize the results in their respective study designs and aims, and discuss whether these might impact the claimed safety and/or feasibility.

Examining the aims of the identified investigations lays open that they mostly follow different or no scientific definitions of a feasibility study. The CONSORT 2010 statement defines that randomized pilot and feasibility trials should primarily aim ‘to assess feasibility of conducting the future definitive RCT’^9^. In other words, the paramount goal is to answering the questions of whether a larger RCT can and should be done and, if the answer is yes, how it should be done^9^. In most of the investigated studies, however, neither these questions nor a plan for a subsequent RCT were even mentioned (five out of twelve). In fact, the scope for their feasibility or pilot study is undefined in four out of twelve investigations.

Although limited-efficacy testing is certainly one appropriate area a feasibility study can focus on^10^, the authors often not explicitly indicate the limited nature of their study but might instead be overly optimistic with their conclusion on the effectiveness. However, this does not necessarily indicate an author’s scientific inability or even malicious intention. Instead, a more fundamentalistic critique towards the scientific publishing system might be appropriate. Author’s might feel pressured towards overly optimistic interpretations of their results as some scientific journals do not accept feasibility studies. More generally, since studies on publication biases show that it is more likely to publish a study if it demonstrates beneficial effects^36,37^, researchers might feel a constrain to over-interpretation or extending pilot and feasibility studies beyond their defined scope^38^. Therefore, it is important to consider that the available data researchers can find in databases might be incomplete if investigations with an assumed less ‘scientific’ relevance did not make it to the publication stage.

Lastly, pilot and feasibility studies are trials with a limited sample size by design. Although it comes with additional resource burdens, it remains desirable that future studies are executed with larger sample sizes for multiple reasons, e.g. to investigate undesirable side-effects better^8,27^. However, it turns out that low recruitment rates already seem to be a problem even in these small investigations. Problems with recruitment went as far as some trials needing to be stopped before reaching the aimed number of participants. Bryant et al. ^29^ point out that it might be due to the strict eligibility criteria, in regards with a cardio-pulmonary exercise testing, but with only two trials reaching a recruitment time rate over three participants per month this seems to be a common problem which might benefit from further investigation. Another downside of larger studies that need to be considered is that larger studies are not only more costly but since they usually take more time, the enlarged timespan also increases the chance of a change in medical therapy during the investigation, which might contradict the inclusion criteria.

Besides the main focus of this systematic review of answering whether or not exercise during treatment is feasible and/or safe

two important key take-aways were drawn from analyzing the investigated studies. The first one addresses both the logistics when carrying out the exercise sessions and safety of the patient.. It is important to recall that exercise sessions can also be carried out in a hospital room with limited space and limited equipment ^33^ The complexity and difficulty of implementing exercise interventions does not only come from the in-hospital setting but additionally that patients sometimes are not allowed to leave their room due to safety concerns. Therefore, it cannot be stressed enough that the hygiene of the used equipment is very important, due to a higher vulnerability to infections and the patients’ therapy-weakened immune system. Additionally, the presence of chest or arm ports or even intravenous lines and poles, which cannot be disconnected during training, demand a well-planned and also flexible exercise protocol to be adjustable for the individual situation^24,27^.

The second insight promises to enhance the outcome quality. Outcome quality might be increased by adding strategies found in health psychology like cognitive-behavioral tools and self-monitoring of exercise^23^, as it might lead to an improvement of adherence numbers. Additionally, the involvement and education of patients and family members in the program and about potential benefits might also enhance motivation^23,33^.

While this study brought to light that exercise appears to be feasible and safe for the population under investigation, it also faces three limitations. First, the title and abstract screening was only conducted by one author, which might have introduced unintended consequences. In future revisions of this review a second author should participate in the initial screening. Second, no quality assessment was conducted. Future investigations might want to find a way to compare and assess the quality between studies of different types (RCT vs feasibility study vs pilot study). Lastly, this review could have been even more focused on AML patients only. Instead, similar diseases were included, which lead to a richer foundation for the review but may led to less precise results.

In this systematic review we summarized the literature regarding the feasibility of exercise training in inpatient patients diagnosed with hematological cancer until April 2022. Across all publications, researchers document the safety and feasibility of exercise interventions. Nevertheless, recruitment rates need improvements to succeed in higher numbers of participants. Additionally, a higher recruitement rate might ultimately reduce the overall costs of a feasibility or pilot study due to time savings when a target sample size is met earlier. One possible option, which appears to be rarely exerted, might be to implement multi centered approaches. Lastly, to increase the overall quality of studies and help evaluating the effectivity of exercise programs and optimizing the exercise regimes, authors should report methodological details for both the planned and executed investigations more elaborately.

References

1. Deschler B, Ihorst G, Platzbecker U, et al. Parameters detected by geriatric and quality of life assessment in 195 older patients with myelodysplastic syndromes and acute myeloid leukemia are highly predictive for outcome. *Haematologica*. 2013;98(2):208-216. doi:10.3324/haematol.2012.067892

2. de Lima M, Anagnostopoulos A, Munsell M, et al. Nonablative versus reduced-intensity conditioning regimens in the treatment of acute myeloid leukemia and high-risk myelodysplastic syndrome: dose is relevant for long-term disease control after allogeneic hematopoietic stem cell transplantation. *Blood*. 2004;104(3):865-872. doi:10.1182/blood-2003-11-3750

3. Erba HP. Prognostic factors in elderly patients with AML and the implications for treatment. *Hematol Am Soc Hematol Educ Program*. Published online 2007:420-428. doi:10.1182/asheducation-2007.1.420

4. Redaelli A, Stephens JM, Brandt S, Botteman MF, Pashos CL. Short- and long-term effects of acute myeloid leukemia on patient health-related quality of life. *Cancer Treat Rev*. 2004;30(1):103-117. doi:10.1016/S0305-7372(03)00142-7

5. Schumacher A, Wewers D, Heinecke A, et al. Fatigue as an important aspect of quality of life in patients with acute myeloid leukemia. *Leuk Res*. 2002;26(4):355-362. doi:10.1016/s0145-2126(01)00145-x

6. Schmitz KH, Campbell AM, Stuiver MM, et al. Exercise is medicine in oncology: Engaging clinicians to help patients move through cancer. *CA Cancer J Clin*. 2019;69(6):468-484. doi:10.3322/caac.21579

7. Schneider CM, Hsieh CC, Sprod LK, Carter SD, Hayward R. Cancer treatment-induced alterations in muscular fitness and quality of life: the role of exercise training. *Ann Oncol Off J Eur Soc Med Oncol*. 2007;18(12):1957-1962. doi:10.1093/annonc/mdm364

8. Pahl A, Wehrle A, Kneis S, Gollhofer A, Bertz H. Feasibility of whole body vibration during intensive chemotherapy in patients with hematological malignancies - a randomized controlled pilot study. *BMC Cancer*. 2018;18(1):920. doi:10.1186/s12885-018-4813-8

9. Eldridge SM, Chan CL, Campbell MJ, et al. CONSORT 2010 statement: extension to randomised pilot and feasibility trials. *BMJ*. 2016;355:i5239. doi:10.1136/bmj.i5239

10. Bowen DJ, Kreuter M, Spring B, et al. How we design feasibility studies. *Am J Prev Med*. 2009;36(5):452-457. doi:10.1016/j.amepre.2009.02.002

11. Thabane L, Ma J, Chu R, et al. A tutorial on pilot studies: the what, why and how. *BMC Med Res Methodol*. 2010;10:1. doi:10.1186/1471-2288-10-1

12. Knips L, Bergenthal N, Streckmann F, Monsef I, Elter T, Skoetz N. Aerobic physical exercise for adult patients with haematological malignancies. *Cochrane Database Syst Rev*. 2019;1(1):CD009075. doi:10.1002/14651858.CD009075.pub3

13. Xu W, Yang L, Wang Y, Wu X, Wu Y, Hu R. Effects of exercise interventions for physical fitness, fatigue, and quality of life in adult hematologic malignancy patients without receiving hematopoietic stem cell transplantation: a systematic review and meta-analysis. *Support Care Cancer Off J Multinatl Assoc Support Care Cancer*. 2022;30(9):7099-7118. doi:10.1007/s00520-022-07029-y

14. AlJohi AA, Aljehani GH, AlSaeed SA, Alhoqail H, Mohammed J, Madi SM. Evidence-based exercises intervention in adults diagnosed with Lymphoma. *Saudi Med J*. 2022;43(5):441-450. doi:10.15537/smj.2022.43.5.20210894

15. Moher D, Liberati A, Tetzlaff J, Altman DG. Preferred Reporting Items for Systematic Reviews and Meta-Analyses: The PRISMA Statement. *Ann Intern Med*. 2009;151(4):264-269. doi:10.7326/0003-4819-151-4-200908180-00135

16. Amir-Behghadami M, Janati A. Population, Intervention, Comparison, Outcomes and Study (PICOS) design as a framework to formulate eligibility criteria in systematic reviews. *Emerg Med J*. 2020;37(6):387-387. doi:10.1136/emermed-2020-209567

17. Eldridge SM, Lancaster GA, Campbell MJ, et al. Defining Feasibility and Pilot Studies in Preparation for Randomised Controlled Trials: Development of a Conceptual Framework. *PloS One*. 2016;11(3):e0150205. doi:10.1371/journal.pone.0150205

18. Barisic A, Leatherdale ST, Kreiger N. Importance of Frequency, Intensity, Time and Type (FITT) in Physical Activity Assessment for Epidemiological Research. *Can J Public Health Rev Can Santé Publique*. 2011;102(3):174-175. doi:10.1007/BF03404889

19. Campbell KL, Neil SE, Winters-Stone KM. Review of exercise studies in breast cancer survivors: attention to principles of exercise training. *Br J Sports Med*. 2012;46(13):909-916. doi:10.1136/bjsports-2010-082719

20. Neil-Sztramko SE, Medysky ME, Campbell KL, Bland KA, Winters-Stone KM. Attention to the principles of exercise training in exercise studies on prostate cancer survivors: a systematic review. *BMC Cancer*. 2019;19:321. doi:10.1186/s12885-019-5520-9

21. Neil-Sztramko SE, Winters-Stone KM, Bland KA, Campbell KL. Updated systematic review of exercise studies in breast cancer survivors: attention to the principles of exercise training. *Br J Sports Med*. 2019;53(8):504-512. doi:10.1136/bjsports-2017-098389

22. Bland KA, Neil-Sztramko SE, Zadravec K, et al. Attention to principles of exercise training: an updated systematic review of randomized controlled trials in cancers other than breast and prostate. *BMC Cancer*. 2021;21(1):1179. doi:10.1186/s12885-021-08701-y

23. Alibhai SMH, Durbano S, Breunis H, et al. A phase II exercise randomized controlled trial for patients with acute myeloid leukemia undergoing induction chemotherapy. *Leuk Res*. 2015;39(11):1178-1186. doi:10.1016/j.leukres.2015.08.012

24. Alibhai SMH, O’Neill S, Fisher-Schlombs K, et al. A clinical trial of supervised exercise for adult inpatients with acute myeloid leukemia (AML) undergoing induction chemotherapy. *Leuk Res*. 2012;36(10):1255-1261. doi:10.1016/j.leukres.2012.05.016

25. Walters SJ, Henriques-Cadby IB dos A, Bortolami O, et al. Recruitment and retention of participants in randomised controlled trials: a review of trials funded and published by the United Kingdom Health Technology Assessment Programme. *BMJ Open*. 2017;7(3):e015276. doi:10.1136/bmjopen-2016-015276

26. Harris LK, Skou ST, Juhl CB, Jäger M, Bricca A. Recruitment and retention rates in randomised controlled trials of exercise therapy in people with multimorbidity: a systematic review and meta-analysis. *Trials*. 2021;22(1):396. doi:10.1186/s13063-021-05346-x

27. Battaglini CL, Hackney AC, Garcia R, Groff D, Evans E, Shea T. The effects of an exercise program in leukemia patients. *Integr Cancer Ther*. 2009;8(2):130-138. doi:10.1177/1534735409334266

28. Baumann FT, Zimmer P, Finkenberg K, Hallek M, Bloch W, Elter T. Influence of endurance exercise on the risk of pneumonia and fever in leukemia and lymphoma patients undergoing high dose chemotherapy. A pilot study. *J Sports Sci Med*. 2012;11(4):638-642.

29. Bryant AL, Deal AM, Battaglini CL, et al. The Effects of Exercise on Patient-Reported Outcomes and Performance-Based Physical Function in Adults With Acute Leukemia Undergoing Induction Therapy: Exercise and Quality of Life in Acute Leukemia (EQUAL). *Integr Cancer Ther*. 2018;17(2):263-270. doi:10.1177/1534735417699881

30. Chang PH, Lai YH, Shun SC, et al. Effects of a walking intervention on fatigue-related experiences of hospitalized acute myelogenous leukemia patients undergoing chemotherapy: a randomized controlled trial. *J Pain Symptom Manage*. 2008;35(5):524-534. doi:10.1016/j.jpainsymman.2007.06.013

31. Duregon F, Gobbo S, Bullo V, et al. Exercise prescription and tailored physical activity intervention in onco-hematology inpatients, a personalized bedside approach to improve clinical best practice. *Hematol Oncol*. 2019;37(3):277-284. doi:10.1002/hon.2576

32. Elter T, Stipanov M, Heuser E, et al. Is physical exercise possible in patients with critical cytopenia undergoing intensive chemotherapy for acute leukaemia or aggressive lymphoma? *Int J Hematol*. 2009;90(2):199-204. doi:10.1007/s12185-009-0376-4

33. Klepin HD, Danhauer SC, Tooze JA, et al. Exercise for older adult inpatients with acute myelogenous leukemia: A pilot study. *J Geriatr Oncol*. 2011;2(1):11-17. doi:10.1016/j.jgo.2010.10.004

34. Streckmann F, Kneis S, Leifert JA, et al. Exercise program improves therapy-related side-effects and quality of life in lymphoma patients undergoing therapy. *Ann Oncol*. 2014;25(2):493-499. doi:10.1093/annonc/mdt568

35. Wehrle A, Kneis S, Dickhuth HH, Gollhofer A, Bertz H. Endurance and resistance training in patients with acute leukemia undergoing induction chemotherapy-a randomized pilot study. *Support Care Cancer Off J Multinatl Assoc Support Care Cancer*. 2019;27(3):1071-1079. doi:10.1007/s00520-018-4396-6

36. Song F, Parekh S, Hooper L, et al. Dissemination and publication of research findings : an updated review of related biases. *Health Technol Assess*. 2010;14(8):1-220. doi:10.3310/hta14080

37. Sterling TD. Publication Decisions and Their Possible Effects on Inferences Drawn from Tests of Significance--Or Vice Versa. *J Am Stat Assoc*. 1959;54(285):30-34. doi:10.2307/2282137

38. Fanelli D. Negative results are disappearing from most disciplines and countries. *Scientometrics*. 2012;90(3):891-904. doi:10.1007/s11192-011-0494-7

39. Conn VS, Hafdahl AR, Porock DC, McDaniel R, Nielsen PJ. A meta-analysis of exercise interventions among people treated for cancer. *Support Care Cancer Off J Multinatl Assoc Support Care Cancer*. 2006;14(7):699-712. doi:10.1007/s00520-005-0905-5

40. Winters-Stone KM, Neil SE, Campbell KL. Attention to principles of exercise training: a review of exercise studies for survivors of cancers other than breast. *Br J Sports Med*. 2014;48(12):987-995. doi:10.1136/bjsports-2012-091732
